# A neuroethical approach to human life, identity, and liberty of schizophrenic patients

**DOI:** 10.1017/S1092852924000506

**Published:** 2024-12-12

**Authors:** Alberto Carrara

**Affiliations:** 1 Faculty of Philosophy and Neurobioethics Research Group (GdN), Pontifical Athenaeum Regina Apostolorum (APRA), Rome, Italy; 2 UNESCO Chair in Bioethics and Human Rights, Rome, Italy; 3 Faculty of Psychology, European University of Rome (UER), Rome, Italy

**Keywords:** consciousness, neuroethics, neurobioethics, philosophy, psychiatry, psychology, human person

## Abstract

This article presents a comprehensive neuroethical framework that seeks to deepen our understanding of human consciousness and free will, particularly in the context of psychiatric and neurological disorders. By integrating insights from neuroscience with philosophical reflections on freedom and personal identity, the paper examines how various states of consciousness from interoception to self-awareness influence an individual’s autonomy and decision-making capabilities. The discussion utilizes a multidimensional, bottom-up approach to explore how neurobiological processes underlie different levels of conscious experience and their corresponding types of freedom, such as “intero-freedom” related to internal bodily states and “self-freedom” associated with higher self-awareness. This stratification reveals the profound impact of neurological conditions on patients’ freedom of choice and the ethical implications therein. The insights gained from this analysis aim to inform more tailored and effective treatments for psychiatric patients, emphasizing the restoration of autonomy and respect for their inherent dignity. This work underscores the essential unity of the human person through the lens of neuroethics, advocating for healthcare policies that recognize and enhance the personal freedom of those with mental health challenges.

## Introduction

Consciousness and free will are fundamental aspects that define the human experience, serving as crucial pillars that distinguish us in the natural world. Consciousness, more than just awareness, involves a complex interplay of sensory perceptions, thoughts, and emotions that allow individuals to interpret and interact with their environment in uniquely human ways. It is the lens through which we perceive the world and our place within it, facilitating nuanced understandings and deep introspections. This self-awareness is essential not only for survival but also for the pursuit of knowledge, the development of personal identity, and the cultivation of relationships. It enables us to reflect on past actions, conceive future possibilities, and engage in the moral and ethical considerations that underpin society.

Free will, on the other hand, plays a pivotal role in actualizing the potentialities presented by consciousness. It affords individuals the autonomy to make choices that reflect their desires, beliefs, and values. The exercise of free will is critical in shaping one’s destiny and is intimately linked to concepts of responsibility and accountability. In a world where actions have consequences, the ability to choose freely is what empowers people to steer their lives and influence the communities around them. Together, consciousness and free will form the bedrock of human dignity and liberty, underscoring the responsibility we have toward ourselves and others in crafting a meaningful existence. Thus, in contemporary discourse, these elements are not just philosophical abstractions but practical necessities that inform legal, social, and ethical frameworks designed to uphold human rights and foster societal progress.

Neurological diseases and mental illnesses can profoundly impact consciousness, altering the very fabric of how individuals perceive, interact with, and understand their environment. Disorders such as Alzheimer’s disease, schizophrenia, and severe depression can disrupt cognitive functions and emotional stability, leading to changes in awareness and a diminished capacity to respond to external stimuli. As these conditions alter the clarity and continuity of conscious thought, they inherently affect an individual’s ability to make informed and intentional choices. The erosion of consciousness can thus lead to a corresponding reduction in free will, as the afflicted may no longer be able to engage with the world in a way that reflects their true intentions and desires.

This interplay between compromised consciousness and diminished free will raises significant ethical and practical challenges. It questions the extent to which individuals with severe mental impairments are responsible for their actions. In legal contexts, this can affect judgments regarding culpability and consent, necessitating a nuanced approach that considers the impact of such illnesses on free will. Similarly, in healthcare, understanding the influence of these conditions on autonomy is crucial for ethical decision-making, particularly regarding treatment and care. Ultimately, acknowledging how neurological diseases and mental illnesses can affect consciousness and free will is essential for fostering compassionate and just societies that recognize the vulnerabilities of their members and strive to support them appropriately.

This exploration of consciousness and free will builds upon and revises previous scholarly proposals by the author in 2018, 2021, and 2023, which articulated a model of human conscious stratification (The author of this contribution published in Italian and English).^1^ ^,^^2^ ^,^^3^ The current discussion enriches these earlier frameworks by introducing a parallel stratification of human freedom, thereby creating a more holistic view of the human experience. This dual stratification approach offers a nuanced perspective that recognizes the layered complexities of both consciousness and free will. It posits that just as consciousness can be viewed as operating at multiple levels—from basic sensory awareness to higher-order reflective thinking—so too can free will be understood as varying in its expression depending on cognitive capabilities, external constraints, and internal psychological states.

This refined model underscores the dynamic interplay between an individual’s level of conscious awareness and the degree of freedom they possess in making decisions. By mapping these dimensions together, the author provides a more comprehensive framework that accounts for the variations in human autonomy and cognitive experience seen across different mental states and in the presence of neurological disorders or mental illnesses. This approach not only advances theoretical understandings in psychology and neuroscience but also has practical implications for fields such as ethics, law, and healthcare. It prompts a reconsideration of how society measures responsibility and autonomy, particularly for those whose capacity for free will is compromised, advocating for policies and practices that are more attuned to the real conditions of human freedom and consciousness.

The author’s perspective is rooted in neuroethics, a field that deals with the systematic and informed reflection on the human condition through the lens of neuroscientific evidence and its interpretations (The term “neuro-ethics” dates back to 1973, although its characterization and diffusion has been taking shape and consistency since 2002).^4^ ^,^^5^ This approach emphasizes the ethical, legal, and social implications arising from advancements in neuroscience. By incorporating neuroscientific findings into discussions of consciousness and free will, the author seeks to bridge the gap between empirical data and the philosophical inquiries surrounding human agency and mental integrity. This integration is crucial, as it helps inform debates about the extent to which neurological and psychological conditions can influence personal autonomy and moral responsibility.

Neuroethics, therefore, serves as a pivotal framework for the author’s analysis, providing the tools needed to critically examine how changes in brain function can impact conscious experience and the exercise of free will. By focusing on this intersection, the author proposes a model that is not only scientifically informed but also deeply attuned to the ethical dimensions of human freedom. Such a perspective is essential for developing sensitive and effective policies that accommodate the complexities of human behavior as revealed through neuroscience. This approach ensures that ethical considerations are not overlooked in the rush to apply new scientific knowledge, thereby safeguarding human dignity and promoting a more nuanced understanding of freedom and consciousness in the face of neurological and mental health challenges.

## Multidimensional bottom-up stratification of human consciousness

In discussing consciousness, it is important to recognize that it is a polysemic term, encompassing a broad spectrum of meanings and implications. In the context of the current discourse, the term “consciousness” is explored from the very inception of human life, beginning with our constitution as biological entities. This perspective traces the arc from conception, where consciousness is considered in its nascent biological form, through the intricate process of cellular interactions that progressively build our biological body. This biological viewpoint frames consciousness not merely as a psychological or philosophical phenomenon, but as an emergent property that begins in the very early stages of life, evolving as cells multiply and differentiate, laying the groundwork for later cognitive and perceptual abilities.

This biological foundation of consciousness underlines the interconnectedness of life and consciousness, suggesting that our cognitive experiences and capacities for free will are deeply rooted in our biological origins. By tracing consciousness back to these fundamental biological processes, the discussion expands to consider how our initial cellular development might influence or correlate with later stages of cognitive consciousness and the unfolding of free will. This approach not only highlights the complexity of consciousness as a concept but also emphasizes the continuity from biological entity to sentient being, offering a comprehensive understanding that spans from the microscopic cellular interactions to the macroscopic level of human behavior and cognition.

In the organic structuring of the human body, a complex interplay of DNA activation and epigenetic factors guides the development and functioning of our biological systems. Central to this developmental journey is the emergence of interoception, which can be considered one of the earliest forms of consciousness. Interoception refers to the perception of internal bodily states rather than external events. It encompasses the body’s ability to sense fundamental homeostatic needs such as hunger and thirst, which are critical for survival. This level of consciousness provides continuous feedback about our internal physiological condition, allowing us to maintain balance within our biological systems.

Interoceptive awareness represents a fundamental dimension of consciousness, establishing a kind of continuous contact with our bodily interiority. This intimate connection with the inner workings of our body not only signals our physiological needs but also potentially influences our emotional experiences and decision-making processes, integrating the biological and psychological facets of human life. Understanding interoception as a primary level of consciousness challenges and expands traditional notions of consciousness, which often focus predominantly on higher cognitive functions and external sensory inputs. Thus, interoception underscores a profound aspect of what it means to be human, reflecting a deep, intrinsic link between our biological states and our experiences of self-awareness and agency.

The second level of conscious experience, exteroception, is critical in the development of human sensory awareness. Exteroception refers to the perception of external stimuli through our sense organs, which involves a complex articulation between these organs and their neurological underpinnings. This level of consciousness is essential for interpreting the environment around us, as it encompasses the integration of sensory data—from sight, sound, touch, taste, and smell—into a coherent perceptual experience. This sensory input is processed by various neural pathways, allowing for the detailed recognition and reaction to external events, which is vital for interaction with the world.

The maturation of human sense experience through exteroception is not merely about the passive reception of stimuli but also involves active processes that enhance our understanding and interaction with our surroundings. For instance, the development of depth perception in vision, the differentiation of tones in hearing, or the complexity of taste and smell discrimination, all depend on the sophisticated integration of sensory information by the brain. These capabilities are foundational not only for basic survival but also for higher-level cognitive functions such as learning, memory, and complex decision-making. Thus, exteroception plays a fundamental role in shaping our engagement with the world, enabling us to navigate, manipulate, and ultimately transform our environment in ways that reflect our needs, desires, and knowledge.

The third dimension of consciousness, often encapsulated by the millennia-old concept of “common sense,” or perception integration, is crucial in forming our own internal representation of the world. This level involves the synthesis of diverse sensory inputs into a unified and coherent whole, allowing individuals to integrate and interpret the multitude of stimuli they encounter. Common sense in this context goes beyond mere sensory perception; it involves the cognitive processes of organizing, prioritizing, and making sense of different sensory data to form a comprehensive picture of reality.

This internal representation is not simply a passive aggregation of data but an active construction that enables humans to navigate their environment more effectively. It allows for the identification of objects and situations, understanding their significance, and anticipating potential outcomes. For example, the ability to recognize a face, understand the mood conveyed by a tone of voice, or anticipate the trajectory of a moving object all depend on this complex integration. This capacity forms the basis of practical intelligence and situational awareness, underpinning not only daily survival but also sophisticated human interactions and behaviors. Through this integrated perception, individuals can apply learned knowledge and abstract reasoning to new situations, a testament to the dynamic and adaptable nature of human cognition.

Gerald Edelman, a Nobel Prize-winning neuroscientist, significantly contributed to our understanding of consciousness by delineating what he termed “primary consciousness.” This level of consciousness, according to Edelman, encompasses both exteroception and perception integration, which together allow for the creation of an internal representation or “scene” of reality. Primary consciousness is described as the ability to generate a mental map of external events, providing a perceptual framework on which reasoning and subsequent actions can be based. This model underscores the brain’s remarkable ability to form a coherent, momentary present from diverse sensory inputs, enabling an organism to navigate and respond to its environment effectively.

This foundational form of consciousness is crucial as it represents the preliminary stage necessary for more complex forms of conscious awareness, such as self-reflection, planning, and deliberate choice. By generating an internal scene, primary consciousness allows for immediate, yet simple, awareness of the world without the depth of past memories or future projections involved in higher consciousness. It is a real-time representation that enables adaptive behaviors essential for survival. Thus, exteroception—the sensing of external stimuli—and perception—the integration and interpretation of those stimuli—serve as the pillars of primary consciousness. These processes equip the brain to function not merely as a passive recipient of information but as an active participant in its perception, providing the groundwork upon which all higher cognitive functions are built.

The fourth dimension of consciousness, awareness, extends beyond the primary consciousness of perceiving and integrating sensory information. Awareness refers to a higher level of conscious experience that involves not only recognizing one’s environment but also having self-awareness and the ability to reflect upon one’s own thoughts and feelings. This form of consciousness is often the focus of neurological studies, particularly in clinical settings where altered states of consciousness, such as minimal conscious states (MCS) and vegetative states (VS), are examined.

In MCS, individuals exhibit a partial preservation of conscious awareness. They can demonstrate some behaviors that indicate a limited awareness of self or environment but do so inconsistently. For example, a person in an MCS might be able to follow an object with their eyes or respond to simple commands intermittently, suggesting some level of awareness, yet lacking the full, consistent engagement seen in fully conscious individuals.

Conversely, in a vegetative state, patients show no signs of conscious awareness despite appearing awake; they might open their eyes or exhibit reflexive responses, but they do not exhibit purposeful behavior or awareness of their surroundings. These states are particularly challenging to study and treat because the presence or absence of awareness can profoundly impact medical, ethical, and legal decisions concerning care and treatment.

The exploration of awareness, especially in these altered states, highlights the complex interplay between various brain functions and regions. Understanding awareness involves deciphering the neurological substrates that enable conscious reflection, emotional processing, and self-recognition. It also encompasses studying how these processes are disrupted, providing insight into the resilience and fragility of the human mind. Awareness is not just a passive occurrence but an active, dynamic process that integrates multiple cognitive and affective components, making it a critical area of study in both neuroscience and clinical practice.

The fifth dimension of consciousness, proprioception, refers to the body’s ability to sense its own position, movement, and overall physical orientation without relying on external cues. This internal sense is sometimes described as the “sense of self” in the physical world, providing the necessary feedback for coordinating movements and maintaining balance and posture. Proprioception is a critical component of consciousness because it integrates sensory information from muscles, tendons, and joints with central nervous system inputs to create a continuous internal map of the body’s position in space.

This proprioceptive awareness is fundamental to all physical activities, from basic actions like walking and standing to complex maneuvers in sports and other skilled behaviors. It allows individuals to perform movements smoothly and efficiently without the need to consciously think about each muscle and limb position. For example, proprioception enables a person to touch their nose with their eyes closed or adjust their gait to uneven surfaces without visually monitoring their feet.

In clinical contexts, the study of proprioception becomes particularly important when dealing with conditions that affect motor control and bodily awareness, such as stroke, Parkinson’s disease, or injuries that involve nerve damage. Loss of proprioceptive ability can severely impact a person’s quality of life, leading to challenges in performing everyday tasks and increasing the risk of falls and injuries.

Overall, proprioception is a vital aspect of consciousness that works largely unnoticed but is essential for interacting effectively with the physical environment. It exemplifies how our bodies and minds are intricately linked, contributing to our sense of self and enabling our interaction with the world around us.

In this bottom-up stratification of conscious manifestations, which maps closely to the increasing complexity of human bodily and particularly neuronal structures, a pinnacle is reached when all elements are organically well-integrated, culminating in the highest level of conscious experience: self-awareness, often referred to as phenomenal consciousness. Phenomenal consciousness represents the sophisticated ability to not only perceive and react to the environment but also to have a subjective and introspective experience of those perceptions and reactions—what is often termed the “qualia” of consciousness.

Phenomenal consciousness enables individuals to experience the world not just through sensory input and cognitive reactions but through deeply personal and unique perspectives. This form of consciousness is where sensations are not merely noted, but felt; where thoughts are not only considered but understood as part of the self’s narrative. This level involves an awareness of one’s existence and identity that transcends simple sensory awareness or the processing of external and internal stimuli—it includes an awareness of one’s own mind and its workings.

The development of phenomenal consciousness is a testament to the intricate and highly organized structure of the human brain, particularly its neuronal architecture, which allows for such complex integrations and manifestations of consciousness. This level of consciousness is what enables humans to reflect on their past, plan for their future, and engage in abstract thinking. It represents a significant evolutionary advantage, allowing for levels of creativity, problem-solving, and social interaction that are uniquely human. Thus, in the hierarchy of consciousness as understood in a neuroscientific context, self-awareness or phenomenal consciousness stands as the most intricate and richly human of all conscious states, encapsulating the full depth and breadth of what it means to be “conscious.”

Indeed, in this multidimensional stratification of consciousness, we can see how it constitutes a privileged place of unity within the human person, profoundly shaping personal identity. Each layer of consciousness, from the most basic perceptions of proprioception and interoception to the sophisticated realms of exteroception and phenomenal consciousness, contributes to a cohesive sense of self. This integration of various conscious experiences provides a continuous internal narrative that is central to personal identity.

Consciousness is not merely a passive recipient of sensations or a simple processor of inputs; it is an active, dynamic force that assembles our thoughts, feelings, perceptions, and memories into a coherent self-image. Through this unification, individuals are able to perceive themselves as distinct entities with unique histories, desires, and future aspirations. The various dimensions of consciousness interlock to create a continuous experience of being, anchoring an individual’s identity over time despite changes in environment or physical state.

This conceptualization of consciousness highlights its role in not just surviving but thriving through the maintenance of a stable yet adaptable identity. It allows individuals to navigate complex social landscapes, reflect on their place in the world, and make meaningful choices that reflect their values and beliefs. In essence, the layered complexity of consciousness is what enables the rich tapestry of human experience, grounding each person’s identity in a continuously evolving but fundamentally integrated perception of self.

## Multidimensional bottom-up stratification of human freedom

The profound connection between consciousness, agency, and free will is elegantly captured in the words of Rita Levi-Montalcini, who highlighted how consciousness serves as a bridge linking our sense of self with our experiences.^6^ This connection is crucial as it empowers us to comprehend our existence as thinking entities, thus grounding us in responsibility for our actions. Consciousness does not merely enable awareness but facilitates a deeper understanding of the implications of our actions, reflecting the core human capacity for choice and moral responsibility.

This synthesis underscores the integral role that consciousness plays in the exercise of free will. By allowing us to evaluate and choose among different options, consciousness imbues our actions with intentionality and purpose. It is this ability to deliberate and make informed choices that form the basis of personal responsibility. As such, consciousness is not just a passive state but an active process that engages with the events of the world and our possible actions within it. Through this dynamic interplay, individuals are not only aware of their actions but also possess the capacity to shape their consequences, a testament to the profound ethical dimension of human consciousness as articulated by Levi-Montalcini. This makes consciousness a foundational element in the development of personal identity and moral agency, illustrating its central role in our lives as both a cognitive and ethical force.

First and foremost, choice (in latin, *electio*) is an act of the will, or human appetitive (tendential) power, realized through a movement toward what is perceived as good, namely a chosen good. The notion of “chosen” presupposes a comparative judgment between alternatives, a preference that precedes and indeed becomes an integral part of the choice itself. This choice is predicated on what might be termed “advice,” that is, a judgment (or a series of reasoning) aimed at determining a course of action. This advice reflects self-consciousness.

From a neuroscientific perspective, it is clear that a “chain” of reasoning underlies any considered decision. Furthermore, these judgments are grounded in their physical capacity to manifest and process, specifically through integration in neuronal structures, particularly in cortical networks.

Similarly, as we have discussed with consciousness, there emerges a gradual bottom-up development for free will manifested in choice. This can be seen as parallel and reflective of consciousness. Just as consciousness is built from the ground up—starting with basic sensory levels like interoception and exteroception, through to perception integration (common sense), awareness, and proprioception, culminating in the highest level of integrative judgment, which is self-aware and self-conscious—so too is the development of free will and its associated responsibility. This multidimensional stratification mirrors the stratification of consciousness and highlights the role of many unconscious processes in influencing our choices.

In this framework, each level of conscious experience has a corresponding dimension of freedom, illustrating a complex interplay between our internal states and our capacity for choice. Interoception, which is the awareness of internal bodily states, corresponds to what can be termed “intero-freedom.” This is the freedom to respond to internal cues such as hunger and pain, guiding decisions that meet basic bodily needs.

Similarly, exteroception—the sensory perception of the external world—corresponds to “extero-freedom,” which involves the freedom to interact with and respond to external stimuli. This type of freedom enables us to navigate and adapt to our environment effectively.

At the level of integrated perception, or what is commonly known as common sense, there arises “integrative freedom.” This represents the ability to synthesize information from various sources, making informed decisions that reflect a holistic understanding of our circumstances.

The level of awareness, which includes a conscious recognition of one’s environment and self, correlates with “aware freedom.” This freedom is characterized by the capacity to reflect on one’s situation and choices, providing a deeper layer of decision-making that considers personal values and ethical implications.

Proprioception, the sense of the relative position of one’s own parts of the body and strength of effort being employed in movement, leads to “proprio-freedom.” This is the freedom to control one’s movements and physical actions with precision, vital for skilled and deliberate activities.

Finally, self-awareness or phenomenal consciousness, where one is fully aware of oneself as a distinct entity with complex thoughts and emotions, corresponds to “self-freedom.” This ultimate form of freedom manifests in the capacity for human phenomenal free action through choices that are deeply reflective, fully considered, and aligned with one’s self-identity and aspirations. This layered approach to consciousness and freedom encapsulates the breadth of human autonomy, reflecting how our internal experiences shape our external choices and actions.

## Life, identity, and liberty of schizophrenic patients

In exploring the complexities of how different conditions affect the relationship between choice and action, it is insightful to consider specific neurological disorders. For instance, patients with apraxia or those suffering from anarchic hand syndrome illustrate distinct disruptions in the pathway from choice to action. Apraxic patients retain their freedom of choice—the apex of the multidimensional pyramid of consciousness and free will. However, due to their pathological condition, they struggle to translate these choices into coordinated actions. In contrast, patients with anarchic hand syndrome can make choices but find their actions do not align with these choices due to involuntary movements of the hand that act independently of their intentions.

Similarly, the condition of schizophrenia profoundly impacts the freedom of choice. Unlike the disorders affecting motor control and action execution mentioned earlier, schizophrenia can interfere with the very capacity to make coherent choices. This disruption occurs due to a variety of symptoms such as delusions, hallucinations, and disorganized thinking, which can cloud judgment and complicate the decision-making process. Patients may find themselves unable to sift through distorted perceptions and thoughts to reach a clear decision, thus affecting their free will at a fundamental level of choice rather than at the level of action execution.

These examples highlight that while the capacity to choose freely and execute those choices can be seen as separate components, they are deeply interconnected. Understanding these nuances is crucial in appreciating the full spectrum of how neurological conditions can influence different aspects of free will, from the formation of intent (choice) to the execution of that intent (action). This knowledge underscores the need for a compassionate and nuanced approach to supporting individuals with such conditions, recognizing the particular ways in which their autonomy and freedom are affected.

In conclusion, the neuroethical stratification proposed in this discussion offers a powerful framework for understanding the conditions of psychiatric patients more profoundly. By examining the different levels at which consciousness and free will can be affected—ranging from basic sensory integration to complex decision-making processes—this model helps to pinpoint where interventions might be most needed. It allows healthcare providers to tailor treatments that address specific impairments in the spectrum of cognitive functions, thereby enhancing the effectiveness of therapeutic approaches.

Moreover, this stratification underscores the essential humanity and inherent dignity of every individual, regardless of neurological or psychiatric conditions. By improving our understanding of how various disorders affect a person’s freedom and decision-making capacity, we can better support their recovery and reintegration into society. This approach not only aims to restore functionality but also to reaffirm the intrinsic value of each patient, recognizing their right to autonomy and a fuller expression of their personal freedom. Such an enlightened perspective is crucial in advancing both the science and the ethics of mental health care, ensuring that patients regain the level of freedom they deserve as beings of infinite dignity.

The principles laid out in this paper offer valuable insights into the complex relationship between consciousness, free will, and schizophrenia. Schizophrenia, characterized by impaired decision-making and distorted perceptions, poses a significant threat to an individual’s sense of autonomy and self-ownership. By understanding the multifaceted nature of free will in schizophrenia, we acknowledge the profound impact this disorder has on patients’ lives and the urgency of addressing their needs through comprehensive and tailored interventions.

The concept of free will is not only a philosophical abstraction but also a fundamental aspect of human dignity, as established by the UNESCO Declaration of Human Rights and Bioethics in 2005 (https://www.unesco.org/en/ethics-science-technology/bioethics-and-human-rights). This paper’s stratification of consciousness and freedom aligns with the declaration’s emphasis on preserving human dignity and autonomy (Art. 3 and Art. 5).

Article 3. Human dignity and human rights.

1. Human dignity, human rights, and fundamental freedoms are to be fully respected.

2. The interests and welfare of the individual should have priority over the sole interest of science or society.

Article 5. Autonomy and individual responsibility.

The autonomy of persons to make decisions, while taking responsibility for those decisions and respecting the autonomy of others, is to be respected. For persons who are not capable of exercising autonomy, special measures are to be taken to protect their rights and interests (https://www.unesco.org/en/legal-affairs/universal-declaration-bioethics-and-human-rights?hub=66535).

It becomes a moral duty to help schizophrenic patients regain authentic ownership of themselves, allowing them to lead a freer and more fulfilling life. This duty extends from pharmacological interventions to encompass psychological and humanistic approaches, fostering an environment that supports the holistic well-being of these individuals.

From a neuroethical standpoint, the pursuit of restoring autonomy to patients with schizophrenia is essential for respecting their inherent dignity (*dignitas infinita*) and promoting their freedom. Is it better to help schizophrenic individuals regain their freedom, or to let them live without autonomy and dignity, languishing in the streets?

The framework outlined in this paper highlights the need for interventions that address the specific impairments affecting free will in psychiatric conditions. By providing compassionate and targeted care, we not only enhance patients’ quality of life but also uphold the core principles of human rights and neuroethics, reaffirming the value of every individual and their right to a meaningful existence.
